# Bridging the gap in pediatric oral health services worldwide: A call for urgent reforms

**DOI:** 10.12669/pjms.42.1.14752

**Published:** 2026-01

**Authors:** Amjad Hussain Wyne, SM Hashim Nainar

**Affiliations:** 1 Amjad Hussain Wyne, Dr Med Dent. President, Pakistan Academy of Pediatric Dentistry, Lahore - Pakistan; 2 S. M. Hashim Nainar, MDSc. Associate Professor, Pediatric Dentistry, Faculty of Dentistry, University of Toronto, Toronto, Canada

**Keywords:** Access to care, Dental education reforms, Oral health policy, Pediatric oral health, Prevention

Oral health is a fundamental component of overall well-being of the individual,^1^ yet pediatric dental health has remained neglected in many parts of the world.^2^ Children who form a substantial proportion of the world’s population, often lack access to adequate dental care due to universal inadequacies in undergraduate dental education, postgraduate dental training, and healthcare infrastructure.^3^ Consequently, even preventable dental diseases continue to afflict millions of children, leading to adverse long-term health consequences.^4^,^5^ The situation warrants a multipronged approach with strategies tailored to individual circumstances.

Many undergraduate dental programs provide only nominal exposure to treating children.^3^ Most dental students graduate with inadequate hands-on clinical experience in pediatric dentistry, often learning only theoretical concepts and manikin simulation operative exercises without the opportunity to practice essential clinical skills in live children with its challenges.^6^ Without adequate training at the undergraduate level, general dentists may lack self-confidence and clinical competence to effectively provide oral health care to children. Reforms must begin at the undergraduate level of dental education. Undergraduate dental curricula should be planned to provide students with articulate hands-on experience in managing pediatric dental patients. This includes compulsory clinical rotations in pediatric dentistry departments, including experience in behavior management techniques, prevention and early diagnosis of pediatric oral diseases. Integrating simulation technology can accentuate training of students but is not a substitute for actual experience with pediatric dental patients.^7^ Dental schools need to fan out their clinical facilities to outreach locations, particularly for senior-year dental students, going out to where patients are available rather than concentrating in a single facility oftentimes in the city core farther away from where patients live.

For young dentists who wish to specialize in pediatric dentistry, the path is often limited by scarce availability of postgraduate training programs. In many countries, the number of available programs in pediatric dentistry is extremely low and disproportionate to the need. This results in a shortage of pediatric dental specialists, further aggravating the lack of access to proper oral health care for children. Without adequate training programs in pediatric dentistry, even interested dentists may be unable to specialize, continuing a vicious cycle wherein pediatric oral health remains underprioritized. National dental councils and universities must mandate increasing postgraduate training opportunities in pediatric dentistry, especially in areas with high child population ratios and limited availability of pediatric dental specialists. Financial inducements such as scholarships and educational loan forgiveness can encourage dental graduates to specialize in pediatric dentistry.

In countries where children make up the majority of the population, oral healthcare facilities earmarked for children are inadequate or totally absent. Public dental clinics and hospitals may offer pediatric dental services, but they are often under-resourced, overloaded, or operated by general dentists lacking skills-set to provide optimal dental services to children.^8^ Training general dentists in basic pediatric dental services, and provision of clear referral pathways will ensure that every child receives proper and timely dental treatment.

The lack of child-friendly dental settings can make dental visits emotionally traumatic for children, leading to avoidance of care and deteriorating oral health outcomes. It is a societal need to build and equip pediatric dental clinics that are esthetically child-friendly and provide specialized dental services. In underserved areas with inadequate healthcare resources, incorporating pediatric dental services into existing public health infrastructure such as primary health care centers or general hospitals can expand availability of dental care for children. These facilities should provide curative and preventive services including education of parents on maintaining their children’s oral health. Healthcare settings physically lacking even basic healthcare facilities may prevent piggybacking of pediatric dental services and warrant mobile dental service vans. In remote areas inaccessible to mobile dental vans or with limited seasonal access, pediatric dental services may benefit from tele-dentistry and emulating successful child immunization campaigns using auxiliary healthcare personnel.

Prevention is the most cost-effective strategy in battling childhood dental diseases. Policymakers must introduce initiatives that support oral health education from an early age in schools, through community campaigns aimed at parents and caregivers. Subsidized dental services such as fluoride applications, dental sealants, and early dental visits can reduce the incidence of dental caries in children. Healthcare authorities should invest in monitoring trends and gauge the effectiveness of existing policies.^4^

Neglecting dental care in childhood has broad consequences that could spread beyond the mouth. In severe cases, untreated carious infections can lead to systemic problems, hospitalizations, and even life-threatening conditions.^9^ Poor oral health during childhood often foretells future dental problems in adulthood, continuing a negative cycle of poor health and economic burden on healthcare systems.^10^ Associations exist between oral health and systemic conditions such as diabetes, cardiovascular disease, and respiratory infections.^11^ Given the growing recognition of the oral-systemic health connection, pediatric oral health must be incorporated into general health policies. Pediatricians and general healthcare workers should be skilled in recognizing early signs of dental disease and be able to guide families toward proper dental care. Oral health examinations should become a component of children’s general health check-ups, especially within maternal and child health programs. Oral health care has received limited political attention and resource allocation in the pursuit of universal health coverage especially in low-income countries. There is an urgent need to mobilize financial and human resources and to integrate preventive and public health-oriented strategies.^12^

The situation of contemporary pediatric dentistry across the globe is a mounting public health concern that warrants urgent attention. Consolidation of undergraduate dental education and postgraduate training, increasing access to pediatric dental services, and additional investments in child-friendly healthcare infrastructure are crucial steps toward ensuring that every child has access to optimal oral care. Pediatric oral health must not be a postscript; it should be a central element of overall health policy.
